# Association between COPD and CKD: a systematic review and meta-analysis

**DOI:** 10.3389/fpubh.2024.1494291

**Published:** 2024-12-16

**Authors:** Zexin Liu, Zhimin Ma, Chaowei Ding

**Affiliations:** ^1^Department of Respiratory and Critical Care Medicine, The Second Hospital of Hebei Medical University, Shijiazhuang, China; ^2^Department of Respiratory and Critical Care Medicine, Xiamen Humanity Hospital Fujian Medical University, Xiamen, China

**Keywords:** chronic obstructive pulmonary disease, chronic kidney disease, systematic review, meta-analysis, comorbidity

## Abstract

**Objective:**

Chronic obstructive pulmonary disease (COPD) and chronic kidney disease (CKD) are significant global health issues with a well-established association between the two. This study aims to assess the risk of developing CKD in patients with COPD through systematic review and meta-analysis, and to explore the impact of CKD on the prognosis of COPD patients.

**Methods:**

A total of 23 studies were included in the analysis, comprising 11 studies on the risk of CKD in patients with COPD, 6 studies on the impact of CKD on the short-term all-cause mortality risk of patients with acute exacerbation of COPD (AECOPD), and 6 studies on the impact of CKD on the long-term all-cause mortality risk of COPD patients. The meta-analysis showed that the risk of developing CKD in COPD patients was significantly increased (OR 1.54, 95% CI: 1.28–1.84), and CKD significantly increased the short-term all-cause mortality risk in AECOPD patients (OR 1.53, 95% CI: 1.44–1.63) as well as the long-term all-cause mortality risk in COPD patients (OR 1.70, 95% CI: 1.35–2.15).

**Results:**

We searched the PubMed, EMBASE, and Cochrane Library databases in accordance with the PRISMA guidelines, including studies from the inception of the databases through December 31, 2023, to identify research assessing the relationship between COPD and CKD. The quality of the studies was assessed using the Newcastle-Ottawa Scale (NOS). Data were analyzed using either a random effects model or a fixed effects model for the meta-analysis.

**Conclusion:**

This study establishes a significant association between COPD and CKD and reveals the adverse impact of CKD on the prognosis of COPD patients, which may provide important guidance for clinical practice.

**Systematic review registration:**

https://www.crd.york.ac.uk/PROSPERO/.

## 1 Introduction

Chronic obstructive pulmonary disease (COPD) is a global public health challenge, with both its incidence and mortality rates on the rise. According to World Health Organization data, in 2019, there were 213.23 million people globally with COPD. This condition resulted in 3.3 million deaths, accounting for 6% of total global deaths. Notably, 90% of these deaths occurred in low-and middle-income countries, placing a significant burden on national healthcare resources. With the intensification of population aging and the extension of life expectancy, the incidence and mortality rates of COPD are expected to continue to increase (1). In addition to progressive lung function impairment, COPD patients often experience systemic complications, including hypertension, diabetes, lung cancer, pulmonary embolism, and pulmonary hypertension (2–5). Among these complications, chronic kidney disease (CKD) has emerged as a significant comorbidity that poses challenges to public health strategies for the older adult population.

CKD is a progressive kidney disease that leads to a decline in renal function, potentially developing into end-stage renal disease (ESRD), which requires dialysis or kidney transplantation (6). Currently, the estimated global prevalence of CKD is about 10%, and this proportion is expected to continue to rise with the aging global population (7). An increasing number of observational studies have shown that the incidence of CKD is closely related to the prevalence of COPD, and research in this area has gradually attracted widespread academic attention.

A German epidemiological study found that the risk of CKD in COPD patients is significantly higher than in the non-COPD population (8). A case–control study from Taiwan also demonstrated that among the middle-aged and older adult population, the prevalence of CKD in COPD patients increased significantly, with an adjusted hazard ratio of 1.61 (*p* < 0.0001) (9). However, some studies have not observed this phenomenon (10–12).

Previous meta-analyses have discussed the prevalence of CKD in patients with COPD but did not fully consider the impact of confounding factors such as age, gender, comorbidities, and smoking. The prognostic implications of CKD in COPD patients remain unclear (13). This study aims to comprehensively evaluate the association between COPD and CKD, clarify the risk of CKD in patients with COPD, and explore the impact of CKD on the prognosis of these patients through systematic review and meta-analysis. By conducting comprehensive literature searches and strict screenings, we hope to provide more reliable evidence to support clinical practice and a scientific basis for public health strategies in aging societies. We will systematically assess the risk of CKD in patients with COPD, as well as the mortality risks associated with COPD, acute exacerbation of COPD (AECOPD), and CKD, to fully explore the relationship between the two.

## 2 Method

### 2.1 Literature retrieval

This study adhered to the Preferred Reporting Items for Systematic Reviews and Meta-Analyses (PRISMA) guidelines (14). A protocol has been registered with PROSPERO under the registration number CRD42024502706.

We conducted a systematic search of literature published in PubMed, EMBASE, and the Cochrane Library from the inception of these databases to December 31, to identify studies assessing the association between COPD and CKD. The search strategy employed keywords such as “chronic obstructive pulmonary disease” and “chronic kidney disease,” optimized with Boolean operators (see Supplementary Table S1 for details). In addition, we manually searched the reference lists of included studies to identify any potentially missed studies.

### 2.2 Inclusion and exclusion criteria

Inclusion criteria: (1) Studies eligible for inclusion include cohort studies, case–control studies, and cross-sectional studies; (2) Provide relevant data between COPD and CKD, such as COPD-CKD risk, CKD-COPD risk, COPD-CKD mortality risk, and AECOPD-CKD mortality risk; (3) The diagnosis of COPD must be clear and confirmed by pulmonary function testing or COPD diagnosis codes in medical records; (4) CKD diagnosis must be clearly defined and confirmed by renal function tests or CKD diagnostic codes in medical records.

Exclusion criteria: (1) Duplicate publications, non-human studies, non-English literature, case reports, reviews, meta-analyses, and conference papers; (2) If there are multiple publications in the same cohort, select the most recently published data; (3) Full-text literature that cannot be obtained; (4) Observational groups and control groups have other diseases that may lead to poor prognosis (such as hypertension, coronary heart disease, etc.).

### 2.3 Data extraction and quality assessment

Two researchers (Liu and Ma) independently screened the literature abstracts and conducted comprehensive full-text reviews to verify their eligibility for inclusion. In case of dispute, a third independent author (Ding) made the final decision. The following information was extracted from the included studies: author, publication year, country, sample size, study design type, patient baseline characteristics, disease diagnosis methods, and related risk data (HR, RR, OR). We used the Newcastle-Ottawa Scale (NOS) to assess the quality of the studies, considering studies with NOS scores ≥7 as high quality.

### 2.4 Data pooling and analysis

HR, RR, and OR were extracted from the included literature and uniformly quantified as OR (15). A meta-analysis was performed using random-effects or fixed-effects models to assess the mutual impact between COPD and CKD. Forest plots were drawn to visually present the combined results of the studies, and the I^2^ statistic was used to assess the heterogeneity of the included studies. When significant heterogeneity was found (*p* < 0.1 or I^2^ > 50%), the random-effects model was used; otherwise, the fixed-effects model was used (16). When significant heterogeneity was present, subgroup analysis was also conducted to explore the sources of heterogeneity. Sensitivity analysis was performed to detect whether the combined results were significantly affected by a single study, and funnel plots and Egger’s test were used to assess publication bias. All analyses were performed using STATA 15.1 statistical software.

## 3 Results

### 3.1 Literature retrieval

**Figure 1:**
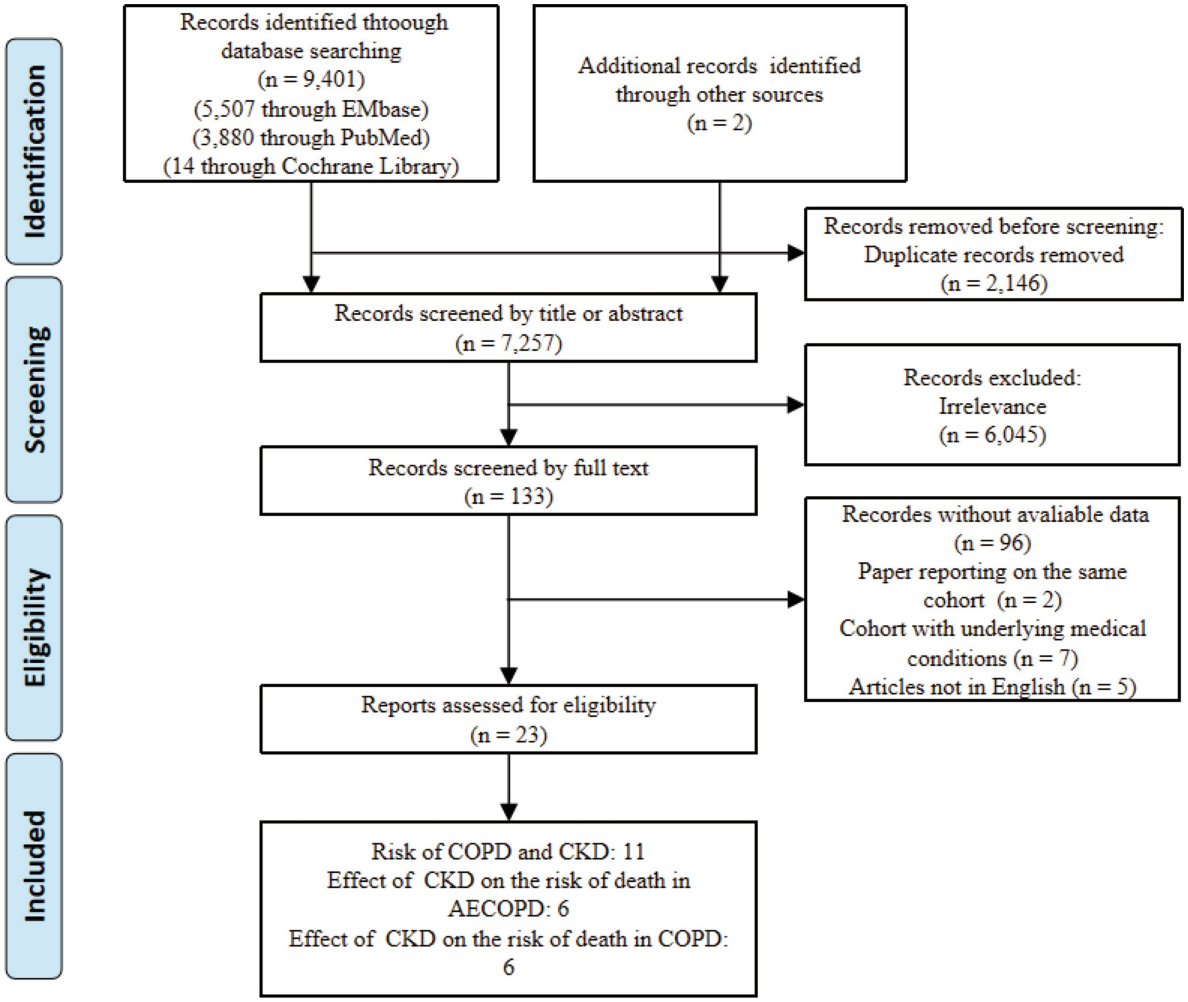
Study selection process diagram.

Using the search strategy, we initially identified 9,401 articles. After excluding 2,146 duplicates, we conducted a preliminary screening of the titles and abstracts of the remaining 7,257 articles, eliminating 6,045 irrelevant studies. We then performed a full-text review of the remaining 133 articles and ultimately identified 23 articles that met the inclusion criteria (refer to Figure 1 for the study selection process).

### 3.2 Basic characteristics of included studies

A total of 23 articles were included, of which 11 examined the risk of CKD in patients with COPD compared to the non-COPD population (8–12, 17–22) (Supplementary Table S2). Six studies assessed the impact of CKD on the short-term all-cause mortality risk in patients with AECOPD (23–28), and another six studies focused on the long-term all-cause mortality risk in COPD patients (Supplementary Table S3) (29–34). These studies encompassed various geographical locations and demographic characteristics, offering a comprehensive perspective. In the 11 studies on the risk of CKD occurrence in COPD, 4 were retrospective cohort studies (8, 9, 19, 20), 2 were case–control studies (10, 21), and 5 were cross-sectional studies (11, 12, 17, 18, 22). Five studies’ data were derived from healthcare databases, with included populations having medical visit records (including outpatient, emergency, and inpatient records) (8–10, 17, 21), four studies’ data were from large-scale population health surveys (11, 12, 18, 22), one study’s data were from a medical center’s health examination population (19), and one study’s data were from outpatients (20). The final results of these studies largely adjusted for risk factors such as age, gender, and comorbidities, but there were still 3 studies that used univariate regression analysis data (8, 10, 20).

Among the studies investigating COPD prognosis, six cohorts reported short-term outcomes for hospitalized patients with AECOPD: four studies assessed in-hospital mortality (23–25, 27), one focused on death within 28 days of admission (26), and one reported mortality within 3 months (28). Furthermore, six cohorts examined the long-term prognosis risk for COPD patients over a duration exceeding 1 year. Among these six cohorts, four studies utilized data from COPD patients enrolled in prospective cohorts established by medical research institutions (29, 31, 32, 34), while the other two studies used data from retrospective cohorts constructed from patient data in medical databases (30, 33).

### 3.3 Quality assessment

The studies included in the research were assessed using the NOS score, with the majority being high-quality studies (score > 6.0), and only one study considered to be of moderate quality (NOS score of 6 points).

### 3.4 Data analysis

#### 3.4.1 The impact of COPD on the risk of CKD

**Figure 2:**
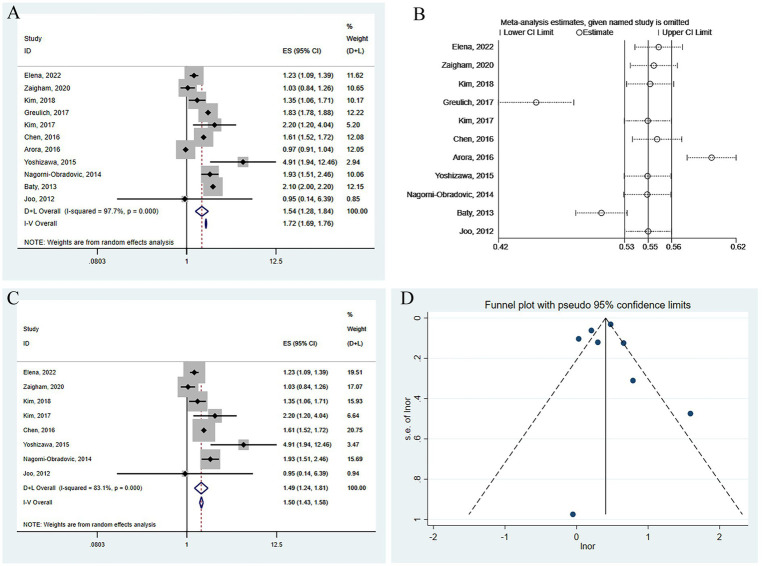
Comprehensive analysis of the risk of CKD occurrence in patients with COPD. **(A)** Forest plot of the risk of CKD occurrence in patients with COPD. **(B)** Sensitivity analysis of the risk of CKD occurrence in patients with COPD. **(C)** Forest plot of the risk of CKD occurrence in patients with COPD after excluding influential studies. **(D)** Funnel plot for publication bias of the risk of CKD occurrence in patients with COPD.

In the 11 studies assessing the risk of CKD in patients with COPD, the odds ratio (OR) for CKD occurrence in COPD patients compared to the normal population was combined using a random effects model: OR 1.54 (95% confidence interval [CI]: 1.28–1.84, I^2^ = 97.7%, *p* = 0.000) (see Figure 2A). A sensitivity analysis was conducted by systematically excluding one study at a time and recalculating the combined OR values for the remaining studies. It was found that three studies Greulich et al. (8), Arora et al. (10), and Baty, 2013 (21) significantly influenced the results (see Figure 2B). In these three studies, the first two did not adjust for any confounding factors in the OR value calculation, and the third only adjusted for age and gender, leading to a possible lower precision of the OR value and a smaller range of the corresponding confidence interval. This may have a significant impact on the final combined results when calculated using the inverse variance method. Therefore, after excluding these three studies, the results were recombined as OR 1.49 (95% CI: 1.24–1.81, I2 = 83.1%, *p* = 0.000) (Figure 2C), which did not qualitatively affect the results and did not reduce the heterogeneity of the combined results. Subgroup analysis was conducted by studying design (cohort or cross-sectional studies), different regions of the study population, sample size, and the analysis methods used in the study results, but no sources of heterogeneity were found (Table 1). A funnel plot was further drawn to observe whether it was symmetrical, and the results did not suggest obvious publication bias (Figure 2D).

**Table 1:** The subgroup analysis for studies about COPD and risk of CKD for all included studies.

Variables	No. of studies	OR (95%CI)	Heterogeneity		Model
			I^2^ (%)	*P*	
Area					
Europe	3	1.33 (0.99–1.80)	87.2	<0.001	Random
Asia	5	1.67 (1.31–2.13)	55.6	0.061	Random
Analysis					
M	7	1.43 (1.19–1.71)	82.9	<0.001	Random
U	1	1.49 (1.94–12.44)	–	–	Random
Sample size					
> 1,000	3	1.44 (1.15–1.81)	97.5	<0.001	Random
NA	1	1.35 (1.06–1.71)	–	–	Random
< 1,000	4	2.20 (1.51–3.21)	30.2	0.231	Fixed
Study designs					
Retrospective cohort	3	1.41 (1.07–1.86)	78.8	0.001	Random
Cross-sectional	4	1.49 (1.24–1.81)	83.1	<0.001	Random

COPD, chronic obstructive pulmonary disease; CKD, chronic kidney diseases; M, Multivariable; U, Univariable; NOS, Newcastle-Ottawa Scale.

#### 3.4.2 Results of short-term all-cause mortality risk in patients with combined CKD in AECOPD

**Figure 3:**
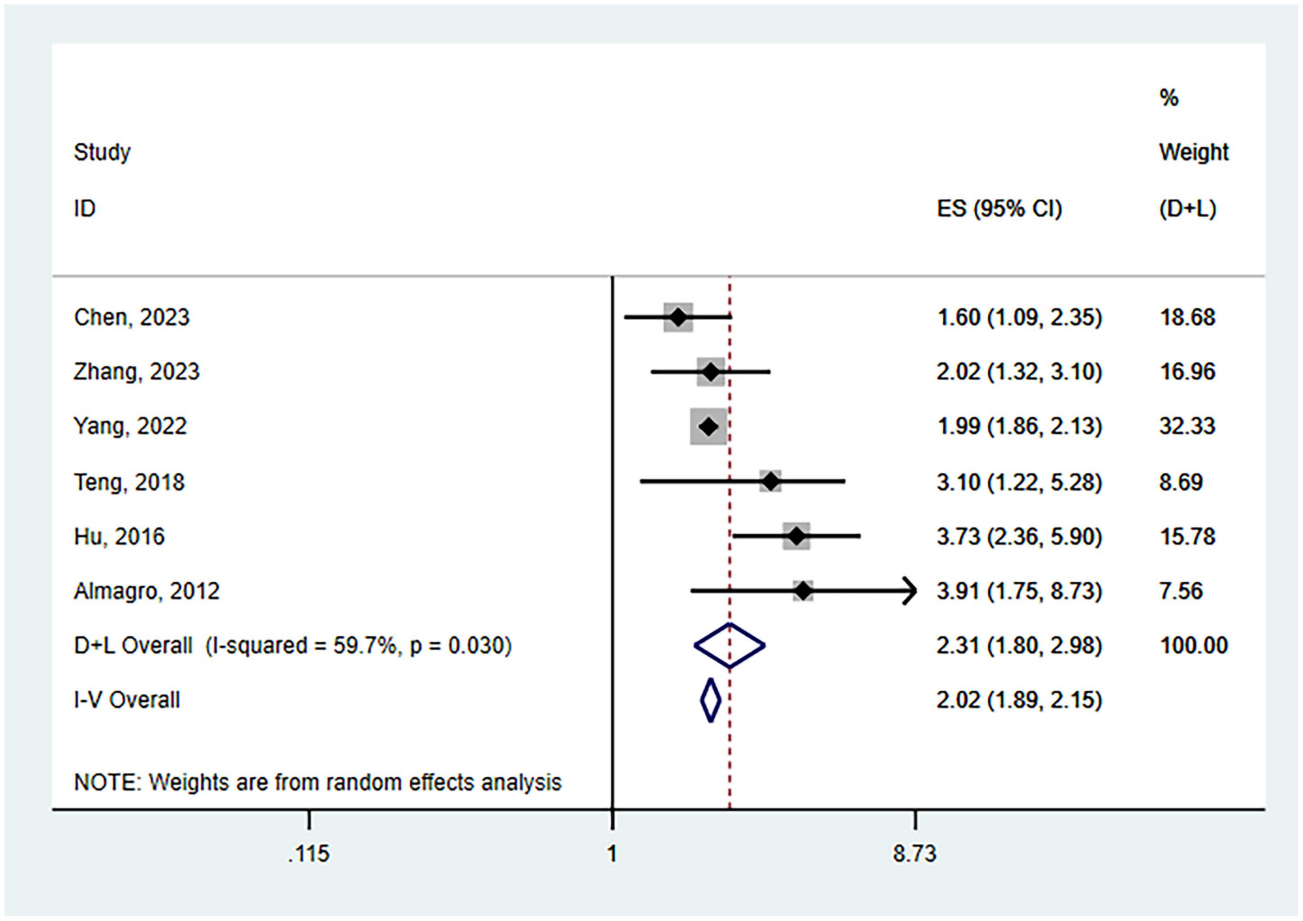
Forest plot of the impact of CKD on short-term all-cause mortality risk in patients with AECOPD.

**Figure 4:**
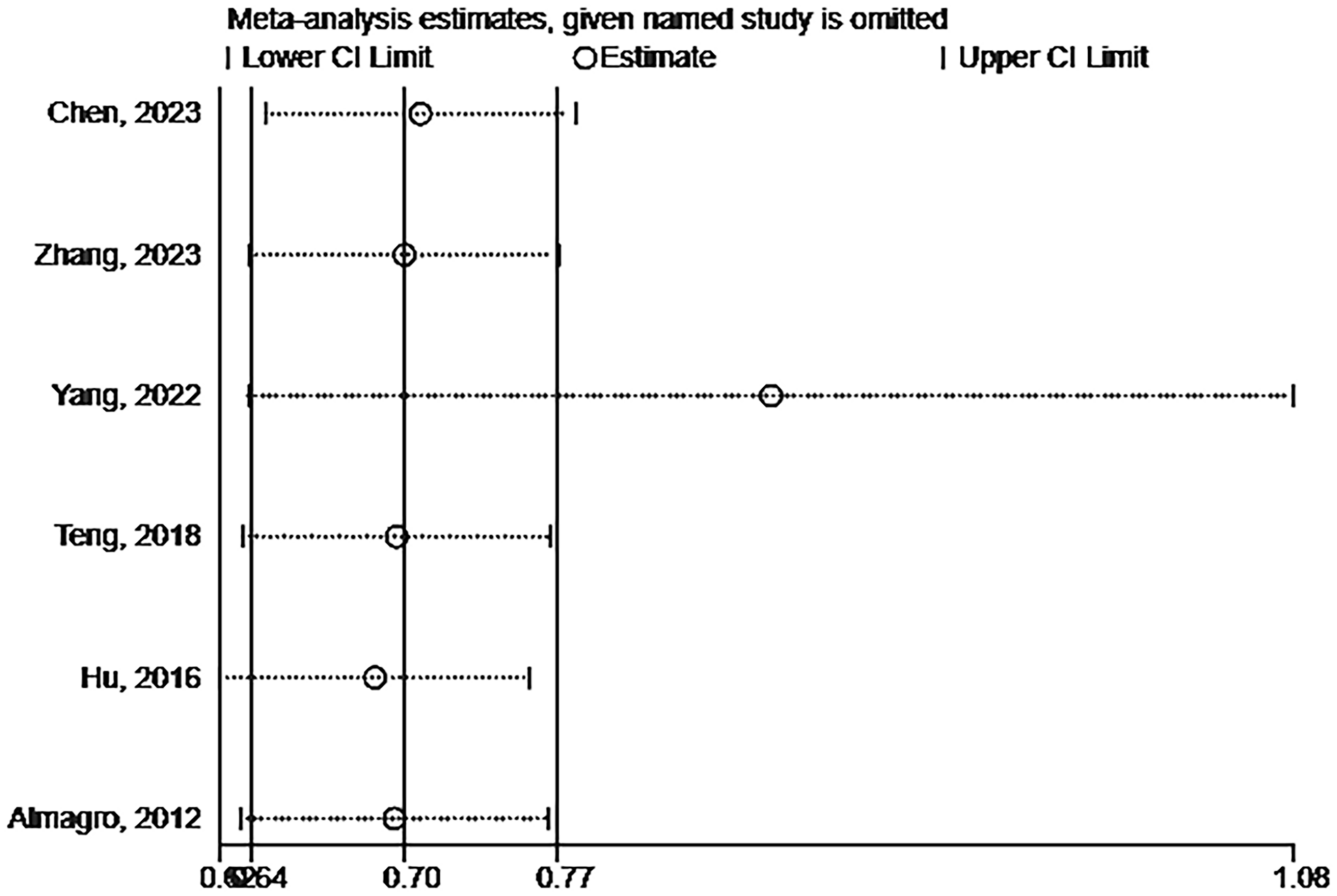
Sensitivity analysis of the impact of CKD on short-term all-cause mortality risk in patients with AECOPD.

**Figure 5:**
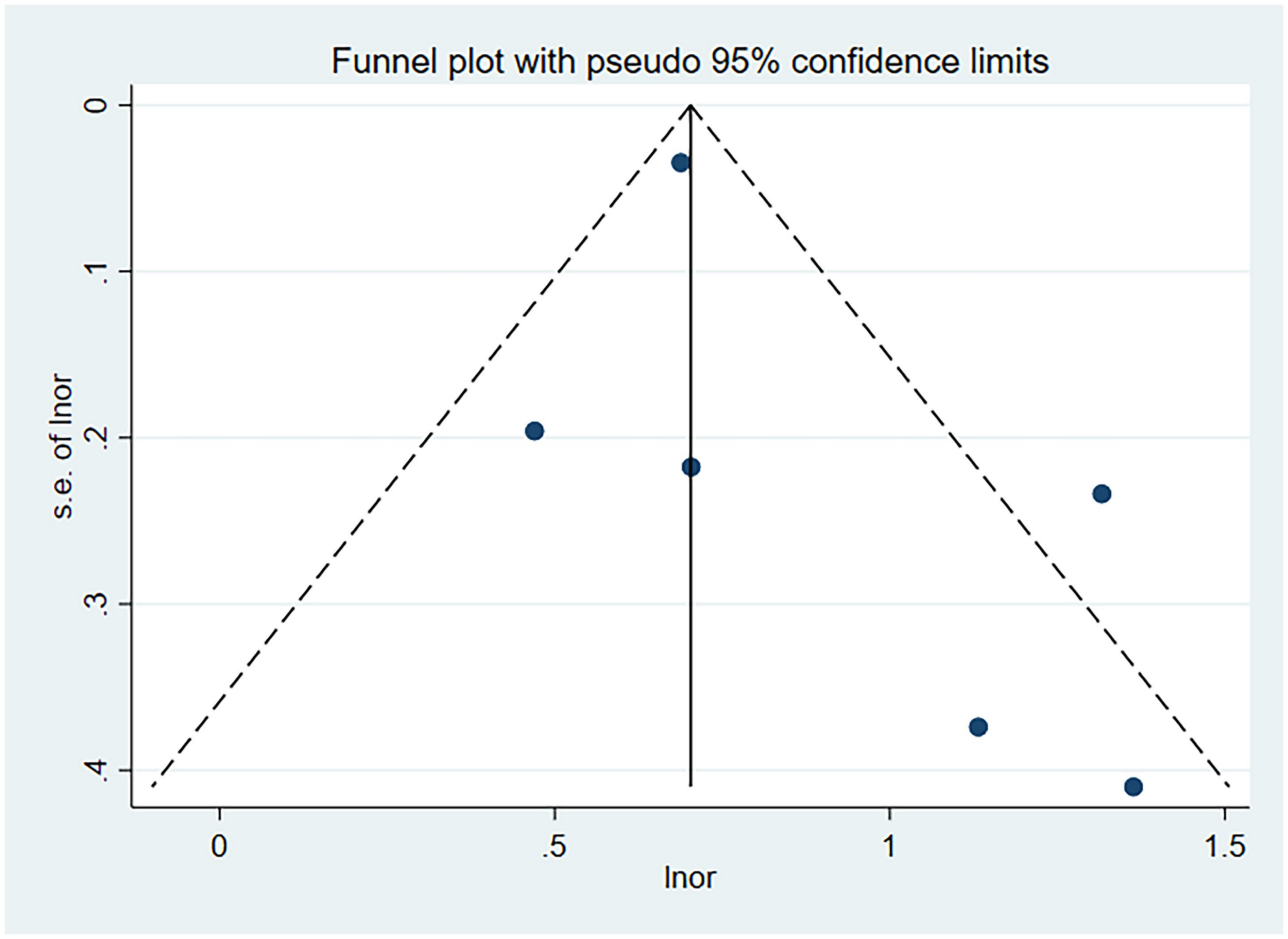
Funnel plot for publication bias of the impact of CKD on short-term all-cause mortality risk in patients with AECOPD.

Six studies assessed the impact of CKD on the short-term all-cause mortality rate in patients with AECOPD. Utilizing a fixed-effects model to combine the short-term all-cause mortality risk data, the results indicated: OR 2.31 (95% CI: 1.80–2.98, I^2^ = 59.7%, *p* = 0.03) (see Figure 3). Sensitivity analysis of the included studies revealed that no single study had a significant impact on the results (Figure 4), suggesting that the combined results are relatively robust. Subgroup analysis was conducted based on study design (cohort or cross-sectional studies), different regions of the study population, sample size, and the analysis methods used in the study results. It was found that sample size may be a significant factor contributing to heterogeneity. When the sample size was categorized as >1,000 or < 1,000, the results showed low heterogeneity, but due to the limited number of studies included, it cannot be confidently explained as the source of heterogeneity (Table 2). The funnel plot appeared symmetrical, which may suggest the presence of publication bias (Figure 5). Further Egger’s test was conducted, and the results did not indicate significant publication bias (Egger’s test = 0.954).

**Table 2:** The subgroup analysis for studies about Risk of short-term all-cause mortality in AECOPD with CKD.

Variables	No. of studies	OR (95%CI)	Heterogeneity		Model
			I^2^ (%)	*P*	
Area					
Europe	1	3.91 (1.75–8.73)	–	–	Random
North America	1	1.60 (1.09–2.35)	–	–	Random
Asia	4	2.43 (1.78–3.32)	64.2	0.039	Random
Analysis					
M	5	2.20 (1.72–2.82)	59.1	0.044	Random
U	1	3.91 (1.75–8.73)	–	–	Random
Sample size					
> 1,000	2	1.78 (1.34–2.36)	0	0.425	Fixed
NA	1	1.99 (1.86–2.13)	–	–	Random
< 1,000	3	3.61 (2.54–5.12)	0	0.895	Fixed
Study cohort					
Retrospective cohort	4	1.99 (1.87–2.13)	44	0.147	Fixed
Prospective cohort	2	2.73 (1.50–4.98)	72.8	<0.055	Random

COPD, chronic obstructive pulmonary disease; CKD, chronic kidney diseases; M, Multivariable; U, Univariable; NOS, Newcastle-Ottawa Scale.

#### 3.4.3 Results of long-term all-cause mortality risk in COPD patients with CKD

**Figure 6:**
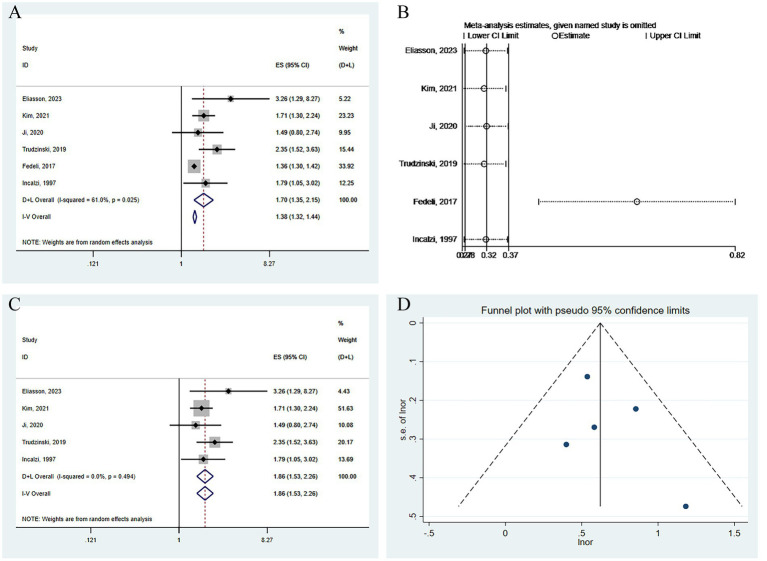
Analysis of the impact of CKD on long-term all-cause mortality risk in patients with COPD. **(A)** Forest plot of the impact of CKD on long-term all-cause mortality risk in patients with COPD. **(B)** Sensitivity analysis of the impact of CKD on long-term all-cause mortality risk in patients with COPD. **(C)** Forest plot of the impact of CKD on long-term all-cause mortality risk in patients with COPD after excluding influential studies. **(D)** Funnel plot for publication bias of the impact of CKD on long-term all-cause mortality risk in patients with COPD.

In the six cohort studies analyzing long-term all-cause mortality risk, we employed a random-effects model to combine the relevant mortality risk results, yielding an OR of 1.70 (95% CI: 1.35–2.15, I^2^ = 61.0%, *p* = 0.025) (see Figure 6A). Sensitivity analysis showed that Fedeli, 2017 (33) had a significant impact on the combination (Figure 6B). After excluding this study, the results were recombined as OR 1.86 (95% CI: 1.53–2.26, I2 = 0.0%, *p* = 0.494) (Figure 6C). The funnel plot was basically symmetrical (Figure 6D), and further Egger’s test was performed, with the result being Egger’s test = 0.410.

## 4 Discussion

This systematic review and meta-analysis investigated the association between COPD and CKD. Our results indicate that patients with COPD have a significantly increased risk of developing CKD, and the presence of CKD significantly increases the mortality risk of patients with COPD. Specifically, CKD not only increases the short-term all-cause mortality risk of hospitalized patients with AECOPD but also significantly increases the long-term mortality risk of patients with COPD.

Both COPD and CKD are chronic diseases with many common etiological factors, such as advanced age (33), smoking (35, 36), and comorbid diseases.

Firstly, with increasing age, the hospitalization rate, length of hospital stay, and mortality rate of patients with COPD all significantly increase. In the process of repeated acute exacerbations, this can cause chronic kidney damage, leading to an increased incidence of CKD (33), so there is a certain connection between advanced age and the incidence of CKD in patients with COPD. Secondly, smoking is also an important common etiological factor for both COPD and CKD, which plays an indirect pathogenic role by damaging the endothelium of pulmonary blood vessels and alveoli (37, 38). It can also damage the endothelial cells of renal microvessels, possibly through regulating oxidative stress responses and activating the sympathetic nervous system, ultimately increasing the risk of CKD (36). In a meta-analysis of 15 prospective studies including a total of 65,064 cases of COPD, the risk of CKD was significantly higher in people with a history of smoking or current smokers compared to non-smokers (39). Lastly, comorbid diseases also play an important role here. Epidemiological survey studies have shown that CKD caused by diabetes accounts for about 30.7% of the total number of CKD cases (7), so among various comorbid diseases, diabetes has the greatest correlation with CKD. In addition to the kidney, the lung is also considered a target organ for diabetic damage. Some cohort studies have shown that patients with COPD are more likely to have diabetes, and diabetic patients are more likely to have COPD than non-diabetic patients (2, 40), which may be caused by various factors, including microvascular changes in alveolar capillaries and pulmonary arterioles caused by diabetes, chronic pulmonary tissue inflammation, and the impact of diabetes on the elastic recoil force of lung tissue and the autonomic nervous regulation of respiratory muscles (41). In addition to diabetes, the relationship between COPD and CKD is also affected by cardiovascular events. Chronic hypoxia and inflammation in patients with COPD aggravate heart dysfunction through various mechanisms (such as increasing cardiac load, vascular damage, and side effects of beta-agonists and other drugs), thereby affecting renal perfusion (42, 43). The presence of cardiovascular events accelerates the progression of CKD, and the incidence of cardiovascular events in patients with CKD is significantly higher than in the general population (44). Therefore, when patients with COPD also have CKD, comorbidities of the cardiovascular system not only affect the patient’s quality of life but also significantly increase the risk of death.

In the literature we included, many studies have statistically adjusted for these common etiological factors, and the final results still have statistical significance. Therefore, there is reason to believe that there are other potential mechanisms that increase the incidence of CKD in patients with COPD.

Firstly, chronic inflammatory response is one of the important mechanisms between COPD and CKD. The characteristic inflammatory response of COPD triggers the release of pulmonary and systemic inflammatory factors, including tumor necrosis factor *α* (TNF-α), interleukin-6 (IL-6), and C-reactive protein (CRP). Studies have shown that these factors are not only highly expressed in patients with COPD but are also closely related to the occurrence and progression of CKD (45). For example, Jingli and others have shown that high levels of CRP are positively correlated with the occurrence and progression of CKD, suggesting that chronic inflammation may lead to a decline in renal function by acting on renal tubular cells and glomeruli (46), in addition, inflammatory-mediated apoptosis and fibrotic processes may also play an important role in patients with coexisting COPD and CKD. Relevant studies have shown that TNF-α causes apoptosis of renal tubular epithelial cells and induces renal interstitial fibrosis by activating the nuclear factor-κB (NF-κB) signaling pathway (47).

Secondly, oxidative stress plays a key role in the interaction between COPD and CKD. The lungs of patients with COPD are affected by oxidative stress caused by chronic inflammation, and the body’s redox state is out of balance, leading to a large amount of reactive oxygen species (ROS) being produced (48). Oxidative stress not only directly damages the structural cells of the lungs but also causes microvascular changes by affecting the hemodynamics of the kidneys. For example, oxidative stress can cause apoptosis and dysfunction of renal endothelial cells, thereby reducing kidney perfusion (49). Some studies have pointed out that oxidative stress may cause vasoconstriction and ischemia by enhancing the activity of nitric oxide synthase, further damaging kidney function (50).

In addition, the accumulation of heavy metals in the body of patients with COPD may be a potential mechanism leading to renal tubular damage. A cross-sectional study from South Korea showed that patients with COPD had increased blood cadmium levels, and regardless of smoking status, the FEV1/FVC ratio in patients with COPD was negatively correlated with blood heavy metal (especially cadmium) levels (51), in addition, another study found that the blood cadmium levels in patients with COPD were also negatively correlated with oxygen saturation during the 6-min walk test (6MWT) (52). Long-term hypoxia caused by COPD can simulate iron deficiency, which may promote the overexpression of DMT-1 in the duodenum, thereby increasing the transport of all divalent cations, including toxic heavy metal cadmium. After absorption, cadmium mainly accumulates in the kidneys and liver, especially cadmium in the kidneys, which further causes damage to renal glomeruli and renal tubules (53). Therefore, there is reason to believe that the process of hypoxia/iron deficiency-DMT-1 overexpression-cadmium overload in patients with COPD may be a possibility for the increased incidence of CKD.

This study acknowledges several limitations that should be considered for future research. Firstly, the majority of the studies included are retrospective cohort or cross-sectional studies, which may lead to incomplete or inaccurate case information. Additionally, the adjustment for comorbidities and other confounding factors varied across studies, potentially resulting in inconsistent adjustments and consequently inconsistent outcomes. Secondly, the diagnosis of COPD in patients primarily relied on diagnostic codes within medical databases, which may not adequately reflect the severity of COPD. Concurrently, the GOLD grade of COPD patients, which significantly influences the risk and prognosis of CKD, was not considered in the multifactorial regression models of most included studies, introducing a possible bias in the final outcomes. Furthermore, the treatment of COPD involves a variety of medications, including antimicrobial drugs and long-acting beta2 agonists (LABA), which could increase the risk of kidney damage. However, the vast majority of the studies did not meticulously document the use of COPD-related medications during the follow-up period, and the impact of these drugs was not adjusted for in the statistical analyses. Therefore, more rigorous clinical studies are warranted to refine these findings and address these limitations.

## 5 Conclusion

In summary, this study, through systematic review and meta-analysis, has established a significant association between COPD and CKD, and has revealed the adverse impact of CKD on both the short-term and long-term prognosis of patients with COPD. Against the backdrop of an aging population, The co-management of COPD and CKD poses significant challenges to public health. Therefore, we recommend that COPD patients undergo regular renal function assessments in clinical management and be managed comprehensively according to the guidelines of the Global Initiative for Chronic Obstructive Lung Disease (GOLD) and Kidney Disease: Improving Global Outcomes (KDIGO) (54) to improve the overall prognosis of patients with COPD.

## Data Availability

The original contributions presented in the study are included in the article/Supplementary material, further inquiries can be directed to the corresponding author.

## References

[1] Safiri S Carson-Chahhoud K Noori M Nejadghaderi SA Sullman MJM Ahmadian Heris J . Burden of chronic obstructive pulmonary disease and its attributable risk factors in 204 countries and territories, 1990-2019: results from the global burden of disease study 2019. BMJ. (2022) 378:e069679. doi: 10.1136/bmj-2021-069679, PMID: 35896191 PMC9326843

[2] Mannino DM Thorn D Swensen A Holguin F. Prevalence and outcomes of diabetes, hypertension and cardiovascular disease in Copd. Eur Respir J. (2008) 32:962–9. doi: 10.1183/09031936.00012408 18579551

[3] Forder A Zhuang R Souza VGP Brockley LJ Pewarchuk ME Telkar N . Mechanisms contributing to the comorbidity of Copd and lung Cancer. Int J Mol Sci. (2023) 24:859. doi: 10.3390/ijms24032859, PMID: 36769181 PMC9918127

[4] Bertoletti L Couturaud F Sanchez O Jimenez D. Pulmonary embolism and chronic obstructive pulmonary disease. Semin Thromb Hemost. (2023) 49:809–15. doi: 10.1055/s-0042-1756190 36108648

[5] Zhang L Liu Y Zhao S Wang Z Zhang M Zhang S . The incidence and prevalence of pulmonary hypertension in the Copd population: a systematic review and Meta-analysis. Int J Chron Obstruct Pulmon Dis. (2022) 17:1365–79. doi: 10.2147/copd.S359873, PMID: 35711174 PMC9196913

[6] Lameire NH Levin A Kellum JA Cheung M Jadoul M Winkelmayer WC . Harmonizing acute and chronic kidney disease definition and classification: report of a kidney disease: improving global outcomes (Kdigo) consensus conference. Kidney Int. (2021) 100:516–26. doi: 10.1016/j.kint.2021.06.028, PMID: 34252450

[7] Global, Regional, and National Burden of Chronic Kidney Disease. A systematic analysis for the global burden of disease study. Lancet. (2017) 395:709–33. doi: 10.1016/s0140-6736(20)30045-3, PMID: 32061315 PMC7049905

[8] Greulich T Weist BJD Koczulla AR Janciauskiene S Klemmer A Lux W . Prevalence of comorbidities in Copd patients by disease severity in a German population. Respir Med. (2017) 132:132–8. doi: 10.1016/j.rmed.2017.10.007, PMID: 29229085

[9] Chen CY Liao KM. Chronic obstructive pulmonary disease is associated with risk of chronic kidney disease: a Nationwide case-cohort study. Sci Rep. (2016) 6:25855. doi: 10.1038/srep25855, PMID: 27166152 PMC4863146

[10] Arora P Gupta A Golzy M Patel N Carter RL Jalal K . Proton pump inhibitors are associated with increased risk of development of chronic kidney disease. BMC Nephrol. (2016) 17:112. doi: 10.1186/s12882-016-0325-4, PMID: 27487959 PMC4973085

[11] Zaigham S Christensson A Wollmer P Engström G. Low lung function and the risk of incident chronic kidney disease in the Malmö preventive project cohort. BMC Nephrol. (2020) 21:124. doi: 10.1186/s12882-020-01758-0, PMID: 32268898 PMC7144045

[12] Joo H Park J Lee SD Oh YM. Comorbidities of chronic obstructive pulmonary disease in Koreans: a population-based study. J Korean Med Sci. (2012) 27:901–6. doi: 10.3346/jkms.2012.27.8.901, PMID: 22876057 PMC3410238

[13] Gaddam S Gunukula SK Lohr JW Arora P. Prevalence of chronic kidney disease in patients with chronic obstructive pulmonary disease: a systematic review and Meta-analysis. BMC Pulm Med. (2016) 16:158. doi: 10.1186/s12890-016-0315-0, PMID: 27881110 PMC5122151

[14] Moher D Liberati A Tetzlaff J Altman DG. Preferred reporting items for systematic reviews and Meta-analyses: the Prisma statement. PLoS Med. (2009) 6:e1000097. doi: 10.1371/journal.pmed.1000097, PMID: 19621072 PMC2707599

[15] Greenland S. Quantitative methods in the review of epidemiologic literature. Epidemiol Rev. (1987) 9:1–30. doi: 10.1093/oxfordjournals.epirev.a036298 3678409

[16] Higgins JP Thompson SG. Quantifying heterogeneity in a Meta-analysis. Stat Med. (2002) 21:1539–58. doi: 10.1002/sim.1186 12111919

[17] Jurevičienė E Burneikaitė G Dambrauskas L Kasiulevičius V Kazėnaitė E Navickas R . Epidemiology of chronic obstructive pulmonary disease (Copd) comorbidities in Lithuanian National Database: a cluster analysis. Int J Environ Res Public Health. (2022) 19:970. doi: 10.3390/ijerph19020970, PMID: 35055792 PMC8775709

[18] Kim MY Boo S Yoo M Lee J Kang NR. Impact of chronic kidney disease among Korean adults with chronic obstructive pulmonary disease. Int Urol Nephrol. (2017) 49:1225–32. doi: 10.1007/s11255-017-1572-4 28386744

[19] Kim SK Bae JC Baek JH Hur KY Lee MK Kim JH. Is decreased lung function associated with chronic kidney disease? A retrospective cohort study in Korea. BMJ Open. (2018) 8:e018928. doi: 10.1136/bmjopen-2017-018928, PMID: 29674361 PMC5914703

[20] Yoshizawa T Okada K Furuichi S Ishiguro T Yoshizawa A Akahoshi T . Prevalence of chronic kidney diseases in patients with chronic obstructive pulmonary disease: assessment based on glomerular filtration rate estimated from creatinine and cystatin C levels. Int J Chron Obstruct Pulmon Dis. (2015) 10:1283–9. doi: 10.2147/copd.S80673 26185434 PMC4500615

[21] Baty F Putora PM Isenring B Blum T Brutsche M. Comorbidities and burden of Copd: a population based case-control study. PLoS One. (2013) 8:e63285. doi: 10.1371/journal.pone.0063285, PMID: 23691009 PMC3656944

[22] Nagorni-Obradovic LM Vukovic DS. The prevalence of Copd co-morbidities in Serbia: results of a National Survey. NPJ Primary Care Respiratory Med. (2014) 24:14008. doi: 10.1038/npjpcrm.2014.8, PMID: 24921714 PMC4373300

[23] Zhang J Qin Y Zhou C Luo Y Wei H Ge H . Elevated bun upon admission as a predictor of in-hospital mortality among patients with acute exacerbation of Copd: a secondary analysis of multicenter cohort study. Int J Chron Obstruct Pulmon Dis. (2023) 18:1445–55. doi: 10.2147/copd.S412106, PMID: 37465819 PMC10351588

[24] Yang C Long J Shi Y Zhou Z Wang J Zhao MH . Healthcare resource utilisation for chronic kidney disease and other major non-communicable chronic diseases in China: a cross-sectional study. BMJ Open. (2022) 12:e051888. doi: 10.1136/bmjopen-2021-051888, PMID: 35027417 PMC8762138

[25] Chen S Shi Y Hu B Huang J. A prediction model for in-hospital mortality of acute exacerbations of chronic obstructive pulmonary disease patients based on red cell distribution width-to-platelet ratio. Int J Chron Obstruct Pulmon Dis. (2023) 18:2079–91. doi: 10.2147/copd.S418162, PMID: 37750166 PMC10518148

[26] Teng F Ye H Xue T. Predictive value of neutrophil to lymphocyte ratio in patients with acute exacerbation of chronic obstructive pulmonary disease. PLoS One. (2018) 13:e0204377. doi: 10.1371/journal.pone.0204377, PMID: 30265703 PMC6161875

[27] Hu G Wu Y Zhou Y Yu Y Liang W Ran P. Cystatin C as a predictor of in-hospital mortality after exacerbation of Copd. Respir Care. (2016) 61:950–7. doi: 10.4187/respcare.04034, PMID: 27072013

[28] Almagro P Cabrera FJ Diez J Boixeda R Alonso Ortiz MB Murio C . Comorbidities and short-term prognosis in patients hospitalized for acute exacerbation of Copd: the Epoc En Servicios De Medicina Interna (Esmi) study. Chest. (2012) 142:1126–33. doi: 10.1378/chest.11-2413, PMID: 23303399

[29] Eliasson G Janson C Johansson G Larsson K Lindén A Löfdahl CG . Comorbid conditions as predictors of mortality in severe Copd - an eight-year follow-up cohort study. Eur Clin Respiratory J. (2023) 10:2181291. doi: 10.1080/20018525.2023.2181291, PMID: 36861117 PMC9970194

[30] Kim Y Kim YJ Cho WK. Effect of multiple comorbidities on mortality in chronic obstructive pulmonary disease among Korean population: a Nationwide cohort study. BMC Pulm Med. (2021) 21:56. doi: 10.1186/s12890-021-01424-7, PMID: 33573635 PMC7879613

[31] Ji Z Hernández-Vázquez J Domínguez-Zabaleta IM Xia Z Bellón-Cano JM Gallo-González V . Influence of comorbidities on the survival of Copd patients according to phenotypes. Int J Chron Obstruct Pulmon Dis. (2020) 15:2759–67. doi: 10.2147/copd.S270770, PMID: 33154636 PMC7608550

[32] Trudzinski FC Alqudrah M Omlor A Zewinger S Fliser D Speer T . Consequences of chronic kidney disease in chronic obstructive pulmonary disease. Respir Res. (2019) 20:151. doi: 10.1186/s12931-019-1107-x, PMID: 31299972 PMC6626422

[33] Fedeli U De Giorgi A Gennaro N Ferroni E Gallerani M Mikhailidis DP . Lung and kidney: a dangerous liaison? A population-based cohort study in Copd patients in Italy. Int J Chron Obstruct Pulmon Dis. (2017) 12:443–50. doi: 10.2147/copd.S119390 28184156 PMC5291454

[34] Antonelli Incalzi R Fuso L De Rosa M Forastiere F Rapiti E Nardecchia B . Co-morbidity contributes to predict mortality of patients with chronic obstructive pulmonary disease. Eur Respir J. (1997) 10:2794–800. doi: 10.1183/09031936.97.10122794 9493663

[35] Chandra D Stamm JA Palevsky PM Leader JK Fuhrman CR Zhang Y . The relationship between pulmonary emphysema and kidney function in smokers. Chest. (2012) 142:655–62. doi: 10.1378/chest.11-1456, PMID: 22459775 PMC3435137

[36] Polverino F Laucho-Contreras ME Petersen H Bijol V Sholl LM Choi ME . A pilot study linking endothelial injury in lungs and kidneys in chronic obstructive pulmonary disease. Am J Respir Crit Care Med. (2017) 195:1464–76. doi: 10.1164/rccm.201609-1765OC, PMID: 28085500 PMC5470750

[37] Wheaton AG Liu Y Croft JB VanFrank B Croxton TL Punturieri A . Chronic obstructive pulmonary disease and smoking status - United States, 2017. MMWR Morb Mortal Wkly Rep. (2019) 68:533–8. doi: 10.15585/mmwr.mm6824a1, PMID: 31220055 PMC6586372

[38] Upadhyay P Wu CW Pham A Zeki AA Royer CM Kodavanti UP . Animal models and mechanisms of tobacco smoke-induced chronic obstructive pulmonary disease (Copd). J Toxicol Environ Health B Crit Rev. (2023) 26:275–305. doi: 10.1080/10937404.2023.2208886, PMID: 37183431 PMC10718174

[39] Xia J Wang L Ma Z Zhong L Wang Y Gao Y . Cigarette smoking and chronic kidney disease in the general population: a systematic review and Meta-analysis of prospective cohort studies. Nephrol Dialysis Transpl. (2017) 32:475–87. doi: 10.1093/ndt/gfw452, PMID: 28339863

[40] Gayle A Dickinson S Poole C Pang M Fauconnot O Quint JK. Incidence of type ii diabetes in chronic obstructive pulmonary disease: a nested case-control study. NPJ Primary Care Respiratory Med. (2019) 29:28. doi: 10.1038/s41533-019-0138-6, PMID: 31308364 PMC6629671

[41] Zhang L Jiang F Xie Y Mo Y Zhang X Liu C. Diabetic endothelial Microangiopathy and pulmonary dysfunction. Front Endocrinol. (2023) 14:1073878. doi: 10.3389/fendo.2023.1073878, PMID: 37025413 PMC10071002

[42] Hesse K Bourke S Steer J. Heart failure in patients with Copd exacerbations: looking below the tip of the iceberg. Respir Med. (2022) 196:106800. doi: 10.1016/j.rmed.2022.106800, PMID: 35306385

[43] Axson EL Ragutheeswaran K Sundaram V Bloom CI Bottle A Cowie MR . Hospitalisation and mortality in patients with comorbid Copd and heart failure: a systematic review and Meta-analysis. Respir Res. (2020) 21:54. doi: 10.1186/s12931-020-1312-7, PMID: 32059680 PMC7023777

[44] Zoccali C Mallamaci F Adamczak M de Oliveira RB Massy ZA Sarafidis P . Cardiovascular complications in chronic kidney disease: a review from the European renal and cardiovascular medicine working Group of the European Renal Association. Cardiovasc Res. (2023) 119:2017–32. doi: 10.1093/cvr/cvad083, PMID: 37249051 PMC10478756

[45] Guo P Li R Piao TH Wang CL Wu XL Cai HY. Pathological mechanism and targeted drugs of Copd. Int J Chron Obstruct Pulmon Dis. (2022) 17:1565–75. doi: 10.2147/copd.S366126, PMID: 35855746 PMC9288175

[46] Gao J Wang A Li X Li J Zhao H Zhang J . The cumulative exposure to high-sensitivity C-reactive protein predicts the risk of chronic kidney diseases. Kidney Blood Press Res. (2020) 45:84–94. doi: 10.1159/000504251, PMID: 31794962

[47] Zhang JQ Li YY Zhang XY Tian ZH Liu C Wang ST . Cellular senescence of renal tubular epithelial cells in renal fibrosis. Front Endocrinol. (2023) 14:1085605. doi: 10.3389/fendo.2023.1085605, PMID: 36926022 PMC10011622

[48] Antus B Kardos Z. Oxidative stress in Copd: molecular background and clinical monitoring. Curr Med Chem. (2015) 22:627–50. doi: 10.2174/092986732205150112104411 25585265

[49] Irazabal MV Torres VE. Reactive oxygen species and redox signaling in chronic kidney disease. Cells. (2020) 9:342. doi: 10.3390/cells9061342, PMID: 32481548 PMC7349188

[50] Carlström M. Nitric oxide Signalling in kidney regulation and Cardiometabolic health. Nat Rev Nephrol. (2021) 17:575–90. doi: 10.1038/s41581-021-00429-z, PMID: 34075241 PMC8169406

[51] Leem AY Kim SK Chang J Kang YA Kim YS Park MS . Relationship between blood levels of heavy metals and lung function based on the Korean National Health and nutrition examination survey iv-V. Int J Chron Obstruct Pulmon Dis. (2015) 10:1559–70. doi: 10.2147/copd.S86182, PMID: 26345298 PMC4531039

[52] Chiu LC Hsu PC Yen TH Kuo SC Fang YF Lo YL . Blood cadmium levels and oxygen desaturation during the 6-minute walk test in patients with chronic obstructive pulmonary disease. Medicina. (2021) 57:–160. doi: 10.3390/medicina57111160, PMID: 34833378 PMC8619611

[53] Cirovic A Denic A Clarke BL Vassallo R Cirovic A Landry GM. A hypoxia-driven occurrence of chronic kidney disease and osteoporosis in Copd individuals: new insights into environmental cadmium exposure. Toxicology. (2022) 482:153355. doi: 10.1016/j.tox.2022.153355, PMID: 36265524

[54] Arif E Medunjanin D Solanki A Zuo X Su Y Dang Y . Β(2)-adrenergic receptor agonists as a treatment for diabetic kidney disease. Am J Physiol Renal Physiol. (2024) 326:F20–9. doi: 10.1152/ajprenal.00254.2023, PMID: 37916289 PMC11194047

