# Supplementary material for: Association between COPD and CKD: a systematic review and meta-analysis
Source: Front Public Health. 2024 Dec 16;12:1494291. doi: 10.3389/fpubh.2024.1494291 (PMC11683117; doi:10.3389/fpubh.2024.1494291)
Supplement: Supplementary file 1 [file Table_1.DOCX]

Supplementary Material

# Supplementary Tables

| Supplementary Table 1. The details of search strategies. | | | |
| --- | --- | --- | --- |
| Database | Search step | Search strategy | Search result |
| PubMed |  |  |  |
|  | #1 | (((((((((((((((((((((Chronic Renal Insufficiencies) OR (Renal Insufficiencies, Chronic)) OR (Chronic Renal Insufficiency)) OR (Kidney Insufficiency, Chronic)) OR (Chronic Kidney Insufficiency)) OR (Chronic Kidney Insufficiencies)) OR (Kidney Insufficiencies, Chronic)) OR (Chronic Kidney Diseases)) OR (Chronic Kidney Disease)) OR (Disease, Chronic Kidney)) OR (Diseases, Chronic Kidney)) OR (Kidney Disease, Chronic)) OR (Kidney Diseases, Chronic)) OR (Chronic Renal Diseases)) OR (Chronic Renal Disease)) OR (Disease, Chronic Renal)) OR (Diseases, Chronic Renal)) OR (Renal Disease, Chronic)) OR (Renal Diseases, Chronic)) OR (ESRD)) OR (end stage renal disease)) OR (renal insufficiency or renal failure) | 386,690 |
|  |  |  |  |
|  | #2 | ((((((((Chronic Obstructive Lung Disease) OR (Chronic Obstructive Pulmonary Diseases)) OR (COPD)) OR (Chronic Obstructive Airway Disease)) OR (Chronic Obstructive Pulmonary Disease)) OR (Airflow Obstruction, Chronic)) OR (Airflow Obstructions, Chronic)) OR (Chronic Airflow Obstructions)) OR (Chronic Airflow Obstruction) | 114,356 |
|  |  |  |  |
|  | #3 | #1 AND #2 | 4,448 |
|  |  |  |  |
|  | #4 | review[Publication Type] | 3,265,662 |
|  |  |  |  |
|  | #5 | #3 NOT #4 | 3,880 |
|  |  |  |  |
|  |  |  |  |
| Embase |  |  |  |
|  | #1 | 'chronic kidney failure'/exp OR 'chronic kidney failure' | 182,556 |
|  |  |  |  |
|  | #2 | 'chronic obstructive lung disease'/exp OR 'chronic obstructive lung disease' | 169,563 |
|  |  |  |  |
|  | #3 | [article]/lim | 29,872,439 |
|  |  |  |  |
|  | #4 | #1 AND #2 AND #3 | 5,616 |
|  |  |  |  |
|  | #5 | review:ti | 847,771 |
|  |  |  |  |
|  | #6 | #4 NOT #5 | 5,507 |
|  |  |  |  |
| Cochrane Library |  |  |  |
|  | #1 | MeSH descriptor: [Pulmonary Disease, Chronic Obstructive] explode all trees | 7,164 |
|  |  |  |  |
|  | #2 | MeSH descriptor: [Stroke] explode all trees | 14,102 |
|  |  |  |  |
|  | #3 | #1 OR #2 | 14 |

| Supplementary Table 2. Characteristics of studies included in prognostic studies. | | | | | | | | | | | |
| --- | --- | --- | --- | --- | --- | --- | --- | --- | --- | --- | --- |
| Author, year | Country | Population,n | Gender,female(%) | Age | Study design | Outcome (follow-up time of death) | COPD type | COPD diagnosis | CKD diagnosis | multi-variates  analysis | NOS |
| Chen, 2023 | USA | 1,346 | 49.48 | 71.26 ± 10.79 | Retrospective cohort | In hospital | AECOPD | Based on ICD codes | Based on ICD codes | NA | 7 |
| Zhang, 2023 | China | 13,431 | 21.26 | NA | Prospective cohort | In hospital | AECOPD | Based on ICD codes | Based on ICD codes | Age, SBP, DBP, pulse rate, hemoglobin, WBC, PaCO2, eosinophil percentage <2%, D- dimer and comorbidities (diabetes, pneumonia, heart failure) | 8 |
| Eliasson, 2023 | Sweden | 241 | 60.17 | NA | Retrospective cohort | 8-9 years | COPD | Spirometry | All Stroke | Age, sex, race/ethnicity, address, median income, number of days admitted, source of admission, hospital size, Charleson Comorbidity Index (CCI) | 6 |
| Yang, 2022 | China | NA | NA | NA | cross-sectional study | In hospital | AECOPD | Based on ICD-10 codes | Based on ICD-10 codes | Age, sex, occupation, type of health insurance, type of admission, intensive care unit stay, and presence of CHD, stroke, hypertension, diabetes, and cancer | 7 |
| Kim, 2021 | Korea | 12,779 | 45.5 | 66.4 ± 11.22 | Retrospective cohort | 6 years | COPD | Based on ICD-10 codes | Based on ICD-10 codes | Age, sex and comorbidities | 7 |
| Ji, 2020 | Spain | 273 | 11 | 67.99 ± 10.62 | Prospective cohort | 68.15 months | COPD | Spirometry | NA | Age, sex, phenotypes and pulmonary function | 8 |
| Trudzinski, 2019 | Germany | 2,274 | 39.4 | 65.0 ± 8.4 | Prospective cohort | 18 months | COPD | Spirometry | eGFR <60 mL/min/1.73 m2 | Age, BMI, sex, packyears, cardiovascular index, and FEV1% pred | 8 |
| Teng, 2018 | China | 904 | 41.92 | NA | Retrospective cohort | 28 days | AECOPD | NA | NA | Age, renal dysfunction, liver dysfunction, heart failure, and upper gastrointestinal bleeding | 8 |
| Fedeli, 2017 | Italy | 27,272 | 39.3 | NA | Retrospective cohort | 5 years | COPD | Based on ICD-9 codes | Based on ICD-9 codes | Age, sex, Charleson Comorbidity Index (CCI) | 7 |
| Hu, 2016 | China | 480 | 25 | NA | Prospective cohort | In hospital | AECOPD | Spirometry (FEV1/FVC <0.70) | renal dysfunction | Age, sex, smoking, PaCO2. PaO2, CHF, CysC, FEV1 predicted | 8 |
| Navaneethan, 2016 | USA | 56,960 | 56.3 | 72.4 ± 11.8 | Retrospective cohort | 3.7 years | COPD | Based on ICD-10 codes | Based on ICD-10 codes | Age, gender, race, CKD stage, diabetes, hypertension, hyperlipidemia, malignancy, insurance, smoking, coronary artery disease, congestive heart failure, cerebrovascular disease, peripheral vascular disease, BMI group, ACE/ARB use, beta blocker use, and statin use, albumin, hemoglobin and current prescription of COPD medications | 8 |
| Almagro, 2012 | Spain | 606 | 10.2 | 72.6 ± 9.9 |  | 3 months | AECOPD | Spirometry (FEV1/FVC <0.70) | NA | NA | 6 |
| Incalzi, 1997 | Italy | 270 | 17 | 67 ± 9 | Prospective cohort | 3.5 years | COPD | Spirometry | NA | Age, sex, ECG signs of ischaemic heart disease, chronic liver disease, length of hospital stay, year of recruitment and history of myocardial infarction | 9 |
| NA: Not available; NOS: Newcastle Ottawa Scale； | | | | | | | | | | | |

| Supplementary Table 3. Characteristics of studies included in this meta-analysis.(Studies about mortality risk) | | | | | | | | | | | |
| --- | --- | --- | --- | --- | --- | --- | --- | --- | --- | --- | --- |
| Author, year | Country | Population,n | Gender,female(%) | Age | Study design | Outcome (follow-up time of death) | COPD type | COPD diagnosis | CKD diagnosis | multi-variates  analysis | NOS |
| Chen, 2023 | USA | 1,346 | 49.48 | 71.26 ± 10.79 | Retrospective cohort | In hospital | AECOPD | Based on ICD codes | Based on ICD codes | NA | 7 |
| Zhang, 2023 | China | 13,431 | 21.26 | NA | Prospective cohort | In hospital | AECOPD | Based on ICD codes | Based on ICD codes | Age, SBP, DBP, pulse rate, hemoglobin, WBC, PaCO2, eosinophil percentage <2%, D- dimer and comorbidities (diabetes, pneumonia, heart failure) | 8 |
| Eliasson, 2023 | Sweden | 241 | 60.17 | NA | Prospective cohort | 8-9 years | COPD | Spirometry | All Stroke | Age, sex, race/ethnicity, address, median income, number of days admitted, source of admission, hospital size, Charleson Comorbidity Index (CCI) | 7 |
| Yang, 2022 | China | NA | NA | NA | Retrospective cohort | In hospital | AECOPD | Based on ICD-10 codes | Based on ICD-10 codes | Age, sex, occupation, type of health insurance, type of admission, intensive care unit stay, and presence of CHD, stroke, hypertension, diabetes, and cancer | 7 |
| Kim, 2021 | Korea | 12,779 | 45.5 | 66.4 ± 11.22 | Retrospective cohort | 6 years | COPD | Based on ICD-10 codes | Based on ICD-10 codes | Age, sex and comorbidities | 9 |
| Ji, 2020 | Spain | 273 | 11 | 67.99 ± 10.62 | Prospective cohort | 68.15 months | COPD | Spirometry | Based on ICD codes | Age, sex, phenotypes and pulmonary function | 8 |
| Teng, 2018 | China | 904 | 41.92 | NA | Retrospective cohort | 28 days | AECOPD | Based on ICD codes | Based on ICD codes | Age, renal dysfunction, liver dysfunction, heart failure, and upper gastrointestinal bleeding | 8 |
| Fedeli, 2017 | Italy | 27,272 | 39.3 | NA | Retrospective cohort | 5 years | COPD | Based on ICD-9 codes | Based on ICD-9 codes | Age, sex, Charleson Comorbidity Index (CCI) | 7 |
| Hu, 2016 | China | 480 | 25 | NA | Prospective cohort | In hospital | AECOPD | Spirometry (FEV1/FVC <0.70) | renal dysfunction | Age, sex, smoking, PaCO2. PaO2, CHF, CysC, FEV1 predicted | 8 |
| Navaneethan, 2016 | USA | 56,960 | 56.3 | 72.4 ± 11.8 | Retrospective cohort | 3.7 years | COPD | Based on ICD-10 codes | Based on ICD-10 codes | Age, gender, race, CKD stage, diabetes, hypertension, hyperlipidemia, malignancy, insurance, smoking, coronary artery disease, congestive heart failure, cerebrovascular disease, peripheral vascular disease, BMI group, ACE/ARB use, beta blocker use, and statin use, albumin, hemoglobin and current prescription of COPD medications | 8 |
| Almagro, 2012 | Spain | 606 | 10.2 | 72.6 ± 9.9 | Retrospective cohort | 3 months | AECOPD | Spirometry (FEV1/FVC <0.70) | Based on ICD codes | NA | 7 |
| Incalzi, 1997 | Italy | 270 | 17 | 67 ± 9 | Prospective cohort | 3.5 years | COPD | Spirometry | Based on ICD codes | Age, sex, ECG signs of ischaemic heart disease, chronic liver disease, length of hospital stay, year of recruitment and history of myocardial infarction | 9 |
| NA: Not available; NOS: Newcastle Ottawa Scale； | | | | | | | | | | | |
